# A novel approach to educate hospitalized cardiovascular disease patients about lifestyle and behavior modifications

**DOI:** 10.1186/s12911-021-01680-x

**Published:** 2021-11-20

**Authors:** Atiyeh Saboktakin, Mohammad Mehdi Sepehri, Roghaye Khasha

**Affiliations:** 1 Faculty of Industrial and Systems Engineering, Tarbiat Modares University, 1411713116 Tehran, Iran; 2 Center of Excellence in Healthcare Systems Engineering, Tarbiat Modares University, 1411713116 Tehran, Iran

**Keywords:** Patient education, Patient engagement, Chronic diseases, Cardiovascular diseases, BPMN, PROMETHEE

## Abstract

**Background:**

Cardiovascular diseases (CVDs) are always considered by healthcare specialists for different reasons, including extensive prevalence, increased costs, chronicity, and high risk of death. The control of CVDs is highly influenced by behavior and lifestyle and it seems necessary to train special abilities about lifestyle and behavior modification to improve self-care skills for patients, and their caregivers. As a result, the development of effective training systems should be considered by healthcare specialists.

**Methods:**

Hence, in this study, a framework for improving cardiovascular patients’ education processes is presented. Initially, an existing training system for cardiovascular patients is reviewed. Using field observations and targeted interviews with hospital experts, all components of its educating processes are identified, and their process maps are drawn up. After that, challenges in the training system are extracted with the aid of in-depth semi-structured interviews with experts. Due to the importance and different influence of the identified challenges, they are prioritized using a Multiple Criteria Decision-making (MCDM) method, and then their root causes were investigated. Finally, a novel framework is proposed and evaluated with hospital experts' help to improve the main challenges.

**Results:**

The most important challenges included high nursing workload and shortage of time, lack of understanding of training concepts by patients, lack of attention to training, disruption of the training processes by the patients’ caregivers, and patient's weakness in understanding the standard language. In identifying the root causes, learner, educator, and educational tools are the most effective in the training process; therefore, the improvement scenarios were designed accordingly in the proposed framework.

**Conclusions:**

Our study indicated that presenting a framework with applying different quantitative and qualitative methods has great potential to improve the processes of patient education for chronic diseases such as cardiovascular disease.

**Supplementary Information:**

The online version contains supplementary material available at 10.1186/s12911-021-01680-x.

## Background

Cardiovascular diseases (CVDs) are a major cause of death and disability in men and women globally [1]. Individuals may be born with a cardiovascular condition or acquire it due to unhealthy patterns of behavior and other factors including adverse social determinants of health [2]. Identification and effective management of these cardiovascular risk factors, especially in high-risk groups such as diabetic patients, are vital [3]. According to the World Health Organization (WHO) report, there are more than 800 risk factors associated with CVDs [4].

Cardiovascular patients who are at high cardiovascular risk (owing to the presence of one or more risk factors such as high blood pressure and diabetes) need early diagnosis and then effective management using consultation and medication [5]. Patient education is a patient-oriented process based on the patient’s needs and the physician’s decisions to make conscious and participatory decisions about the disease, learn self-care skills for patients, and control disease. Patient education encompasses all educational activities related to illness, including therapeutic and hygienic actions, and promoting clinical health training [5].

On the other side, CVDs are one of the chronic diseases. Therefore, in the prevention phase, patient education is essential. At the treatment phase, long-term adherence of patients to medical guidelines leads to more effective disease control and institutionalization of education in patients [6]. Effective management of chronic diseases depends on the ability of patients and their caregivers to undertake self-care items over time [7]. The most important way to achieve this goal is to educate and engage patients [8].

Health education has been addressed by many healthcare specialists, doctors, and researchers, and there are ongoing efforts to develop and improve it [9, 10]. Despite the interest of health centers in empowering patients to perform self-care activities in chronic diseases, they are not mostly satisfied with the outcome of the relevant educational processes [11]. For instance, Boyde, Grenfell [12] conducted a study on 135 hospitalized cardiovascular patients and evaluated patient education results in three levels of knowledge, attitude, and belief. These three indicators were used to assess the effectiveness of the education system. The scores of response to the indicators were very low, indicating the ineffectiveness of patient education.

Many researchers have attempted to identify the challenges in the processes of patient education. Some regard these challenges as systematic failures, and others find them related to the patients’ characteristics and behaviors. For example, age is one of these factors because patients are exposed to various health-education problems in older age [9]. Recently, comorbidity has also become more prevalent with age, makes self-care and patient education increasingly complex [9]. Maloney and Weiss [13] claim that the roots of these phenomena are patients’ lack of attention when transmitting information, the difficulty of confronting the disease, sense of fear, and a large amount of data. Due to the importance of education in the treatment and control phases of chronic diseases such as CVDs, it is crucial that educational processes lead to a series of care and self-care activities that can change the patients’ lifestyle in the long run [7]. Abandoning and changing habits like smoking, inappropriate food habits, and lack of sufficient exercise that have become a part of patients’ behaviors and lifestyle in the long term, are very challenging [7]. Dickson and Riegel [10] showed that doctors’ recommendations for continuous jogging or participation in sports clubs seem strange to most cardiovascular patients, who had no regular exercise program.

On the other hand, according to Riegel and Carlson [14] and Dickson and Riegel [10], misconceptions and lack of awareness among cardiovascular patients are very common. Dickson remarked that many scholars believe that a significant number of patients consider sport as something forbidden for their health. But education is a necessary response to this lack of knowledge and awareness in patients. Health literacy is recognized as a factor, which is very useful in patients’ ability to understand and use health information and guidelines. The Institute of Medicine Committee on Health Literacy has stated that patients with inadequate health literacy have less partnership in decision making and low adherence to their doctors’ treatment plan [4].

Physicians and nurses play a very influential role in the education process of their patients [15]. However, challenges such as lack of enough time and fear of risking the doctor-patient relationship prevent them from developing patient education processes [16]. Arian, Mortazavi [17] studied the barriers to educating patients from the management and nursing perspectives in one Iranian hospital. They identified the most critical challenges as follows:

- lack of nurses’ knowledge and awareness about the principles of patient education, illiteracy of patients, lack of patients’ cooperation, lack of recognition of patients’ educational needs, lack of educational resources, lack of regular planning for patient education, poor health status, lack of known laws for patient education, lack of human resources for education, different sex of educators and patients, patients’ inability to take care of themselves, high nursing workload, and insufficient budget allocation for patient education.

In previous studies, there were shortcomings such as the lack of a complete and comprehensive categorization of the challenges of patient education and engagement, and evaluating their effectiveness on the education process. Besides, researchers that have addressed the root causes of these challenges, just have provided solutions for improving the education system and paid less attention to the root causes in the overall system. The challenges of patient education identified in previous researches are illustrated in Table 1. Every challenge is marked with a code from CH1 to CH29 (CH is an abbreviation of “Challenge”). Also, Table 1 shows supporting evidence (citations) for all challenges.

**Table 1:** Patient education challenges extracted from the review of the literature

Challenges of the CVDs education system		
Human resources-related challenges	Patient-related	Illiteracy **(CH1)** [9, 17]
		Patient's paying less attention to the instructor **(CH2)** [13]
		Fear of too much information **(CH3)** [13]
		A misconception or lack of awareness **(CH4)** [10, 14]
		Difficulty of changing lifestyle **(CH5)** [10, 14]
		Patients' lack of understanding about the severity of the disease **(CH6)** [12]
		Difficulty in memorizing information **(CH7)** [13]
		Confusion because of conflicting information **(CH8)** [10]
		Difficulties in facing the disease **(CH9)** [13, 14]
		Age-related cognitive disorder **(CH10)** [14]
		Lack of patients' cooperation **(CH11)** [14, 17]
		The patients' unfavorable status **(CH12)** [13, 17]
	Medical personnel-related	Lack of personnel’s time and long duration of education classes **(CH13)** [9, 17, 26]
		Lack of nurses’ awareness about patient education principles **(CH14)** [17]
		Risk of weakening patient-doctor relationship **(CH15)** [26]
Society-related challenges	Different culture and language **(CH16)** [9]	
	Old population **(CH17)** [9]	
	Limited health literacy **(CH18)** [4]	
	Comorbidity outbreak **(CH19)** [9]	
System-related challenges	Planning-related	Lack of flexible and dynamic educational plans **(CH20)** [12, 17]
		Relevance of provided education **(CH21)** [13]
		Direct relation between the amount of education and duration of hospitalization **(CH22)** [12]
		Medical advances and increasing amount of transmitted information **(CH23)** [9]
		Uncertainty of patients' understanding of info at the time of discharge **(CH24)** [13]
		Health centers focus on their own desired content (not patients' needs) **(CH25)** [13, 17]
		Lack of recognition of the patients' educational needs **(CH26)** [17]
		Lack of nursing manpower **(CH27)** [17]
		Lack of written educational sources **(CH28)** [17]
		Lack of funding for patient education **(CH29)** [17]

It seems that comprehensive identification of the challenges of the process of educating cardiovascular patients and, as well as improving the education process by presenting a new framework, are the very topics that have not received enough attention in the past researches. Different researches have been done to identify, prioritize, and then analyze the challenges of the patient education process in various health care systems. However, no similar study has been conducted in terms of performing simultaneously the four actions of identifying challenges, prioritizing them, rooting them out, and providing a comprehensive framework to improve the implementation of educating cardiovascular patients.

In addition, there are several methods for prioritizing the challenges. However, in previous similar researches, just the questionnaire tool has been used to survey and rank the experts' opinions. To address the problem, the present study uses one of the multi-criteria decision-making methods for prioritizing challenges after identifying them. The challenges identified must be prioritized because there are too many of them, and they have various backgrounds. Furthermore, not all challenges are important equally, and not all of them have an equal impact on the education system, so their importance must be prioritized. Therefore, to identify the most important challenges and provide improvement scenarios of the education system, prioritization must be performed.

In industrial engineering, studying the process of patient education falls into qualitative studies, and even quality management specialists do not usually pay enough attention to it. A review of the related literature indicates that studies have often been undertaken in the field of nursing or educational management of hospital. Hence, selecting appropriate health educational systems and then examining them using a mix-method approach is a relatively new step in industrial engineering research.

It is noteworthy that this applied research has been done by studying a particular case. The implementation steps of this research have been designed based on a structured, step-by-step problem-solving system to answer the following research questions:

1. What is the as-is process of cardiovascular patient education in hospitals?
2. What are the challenges to educating and engaging patients in an education system?
3. Which of the challenges has more impact on the education process of cardiovascular patients?
4. How does a useful framework of a cardiovascular patient education system address the existing challenges?

## Methods

### Study area

This research was conducted in cooperation with Tehran Heart Center (THC), affiliated with Tehran University of Medical Sciences, which is a teaching, research, and therapeutic hospital. The THC was inaugurated in 2001 with 460 inpatient beds. This hospital is one of the best-equipped diagnostic and therapeutic cardiology centers in the Middle East [18].

### Research methodology

**Fig. 1:**
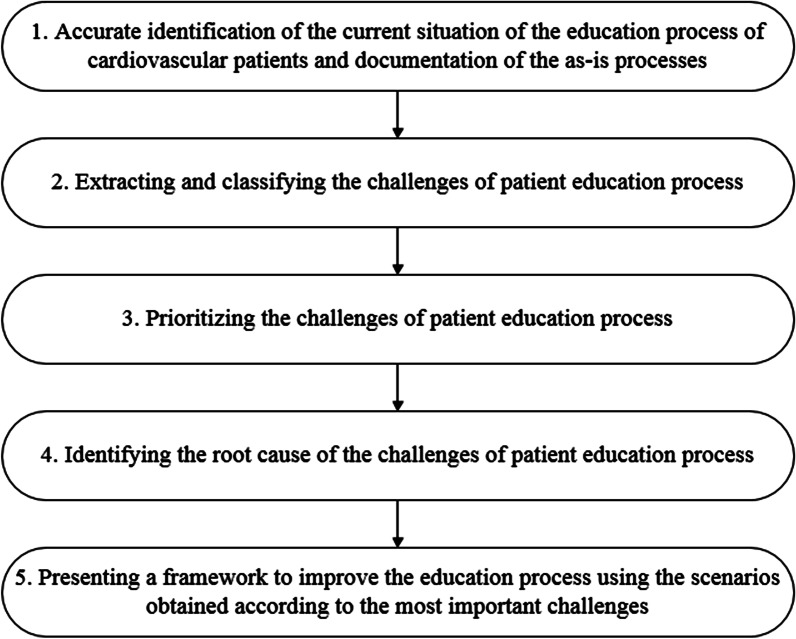
Steps applied in the methodology of the research

The methodology used in the present research is based on a problem solving structured step-by-step system that has been a generalization of the business process management (BPM) method [19] presented in Fig. 1.

In the first stage, the required data were collected using the hospital documentation, field observation, and interviewing the hospital experts to identify the challenges of the education process and engaging the cardiovascular patients. In this phase, we employed the Business Process Model and Notation (BPMN) standards and cross-functional chart to document the education process [19]. BPMN is one of the complete languages of modeling the business processes; for this reason, this symbolic modeling language was used for documenting the education processes in the current study. The implementation of BPMN basically consists of 3 layers:

- Layer 1: perception and understanding the processes,
- Layer 2: improving the processes,
- Layer 3: and mechanizing and optimizing the processes.

In the second stage, the challenges of the patients' education system were extracted through semi-structured in-depth interviews [20] with experts at the hospital and then classified and coded by the researchers. Semi-structured interviews are among the most common types of interviews used in qualitative research. In this type of interview, all interviewees are asked similar questions about the subject under study, but they are free to provide their answers in any way they wish. In this interview, the researcher is responsible for coding and classifying the answers.

**Fig. 2:**
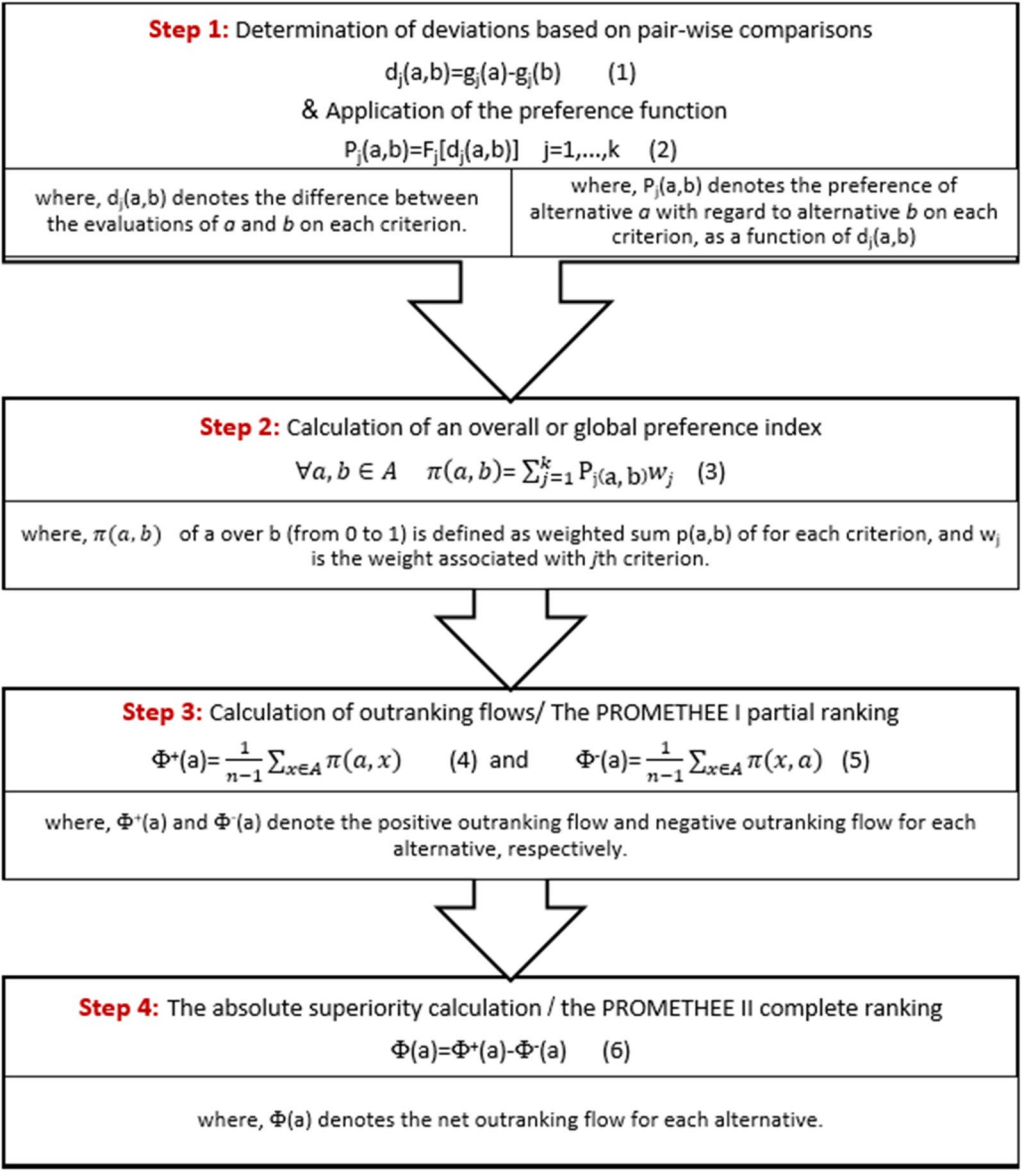
PROMETHEE II steps

Prioritizing the identified challenges is essential because there are too many challenges with varying degrees of importance in this process. As a result, in the third stage, prioritizing the challenges of the patient education system was performed using the Preference ranking organization method for enrichment evaluation (PROMETHEE) method [21]. A review of previous research about prioritizing health-related subjects showed that researchers have used qualitative approaches and questionnaires to evaluate challenges and prioritize them, while in other domains such as industry affiliates, MCDM [22] methods have been used that are powerful tools for ranking. PROMETHEE II method is one of the most complex MCDM methods, which are very accurate. MCDM methods are used to evaluate alternatives and prioritize them based on several criteria. We chose the PROMETHEE II method for prioritizing the challenges due to the type of alternative scoring, simple scoring, and powerful ranking mechanism. Figure 2 illustrates the steps for implementing this method. In the first step of this method, a decision matrix is formed and then normalized. A decision matrix is a matrix for evaluating a number of alternatives (challenges) based on a number of criteria. That is a matrix in which each alternative (challenge) is scored based on a number of criteria. After that, the deviations are determined based on pairwise comparisons of alternatives. In the second step, a relevant preference function is used for each criterion. In the third step, the global preference index is calculated. In the fourth step, positive and negative outranking flows (Φ^+^ and Φ^−^) for each alternative and partial ranking are calculated. In the last step, the net outranking flow for each alternative (Φ) is calculated, and ranking is completed.

After Prioritizing challenges with the PROMETHEE method, in the fourth stage, for the top ten prioritized challenges, the root causes analysis was performed to detect their root causes by the hospital experts. Then improvement scenarios for the education system based on them were suggested.

In the final stage, focusing on the challenges affecting the education system and the outcome of the root cause analysis, a novel framework for improving the education processes was proposed with the hospital experts' help. In the following, the executive stages of the proposed approach and the obtained results are presented.

The results presented in the next section are consistent with the research steps shown in Fig. 1. This research consists of five different steps, and the outputs of each step are presented in headings similar to Fig. 1, respectively.

## Results

### Identifying the current education process based on the BPMN standards

In the first stage, we employed the BPMN standards and cross-functional charts to document the current situation of the education processes of cardiovascular patients (the as-is processes) at the hospital under study. In this hospital, there are three main education processes in the inpatient ward, surgical inpatient ward, and angiography. They include many sub-processes that some of which are not necessarily in the same ward and might be performed by different persons in different stages of patient flow in the hospital. In the present study, a big challenge in presenting the maps of the processes to their owners was the lack of familiarity with the BPMN standards. In this regard, cross-functional charts were used to simplify the process maps. These charts incorporate all information of the processes, including activities, events, and flow sequences. Hence, the cross-functional charts of the three educational groups (inpatient ward, surgical inpatient ward, and angiography) were drawn up using the Visio 2013 software. The drawn maps of the process are summarized in Additional files 1, 2, 3: A.1, A.2, and A.3.

### Identifying the challenges of educating cardiovascular patients

After validating the process maps, semi-structured in-depth interviews were arranged with selected process owners and hospital experts to extract the challenges of each stage of educating patients. In these interviews, the experts were asked to express the challenges of educating and engaging patients according to their experience. Firstly, the interviews were conducted with selected experts, and the responses were compared to the previous ones. If a relatively new topic was raised in each interview's responses, the next new interview would be added so that the information overload takes place. Eight head nurses, three doctors, 18 nurses, and 15 patients were interviewed in different surgical and internal departments in separate sessions. After analyzing the content of the answers, the information extracted from each interview was reviewed by the experts to verify their validity. We have conducted a member check with respondents after we have collected the data and undertaken an initial analysis (respondent feedback after analysis). This involves going back to our respondent group with the initial results and asking, "have we interpreted your responses correctly" and "are our assumptions/conclusions valid"? After final verification, all challenges were classified and then coded into three main groups (Table 2):

1. Challenges related to human resources
2. Challenges related to the work environment and conditions
3. Challenges related to management and system.

**Table 2:** Extracted challenges of the process of patient education

Challenge	Sub-challenge	Code	Challenge	Sub-challenge	Code
Challenges related to human resources (HR)	The patient does not listen carefully (The patient does not follow the educators & does not pay due attention to the educations)	HR1	Challenges related to the work environment and its condition (E)	The educational films provided for the classes are not suitable in terms of duration, quality, and content	E1
	The patient does not understand the meaning of education content	HR2		The patient does not have the training booklet throughout hospitalization	E2
	The patient forgets	HR3		Medical education information is often forgettable to the patient	E3
	The patient is illiterate	HR4		The nurse does not have the time to evaluate the effectiveness of the provided education	E4
	The patient does not understand the standard language	HR5		Change in the patient’s behavior (attitude and belief) is not measurable in the short period of hospitalization	E5
	The patient is not aware of the importance of the educational subject	HR6		The education room or the inpatient ward room is small, and there are not adequate group classrooms	E6
	The patient is not aware of his/her rights	HR7		In crowded classes, temperature and air circulation are not suitable	E7
	The patient does not accept the illness, or the mental problems resulting from the illness decrease the educational effectiveness	HR8		Different candidates are chosen for each individual during the education time by the nurse/physician	E8
	The patient does not consider the information provided by the doctor/nurse as education	HR9		Educational tools, including booklet and brochure, are not interactive	E9
	The patient doesn’t understand the medical terms used by the treatment team	HR10		Educational tools are not used appropriately	E10
	Some of the patient's caregivers request for the same education that has already been given to the patient	HR11		There are no appropriate visual tools for education	E11
	The patient's family does not want her/him to know about the disease or the treatment	HR12		There is no continuous educational platform (covering educational tools or an educator) inside or outside the health center when needed	E12
	The nurse is too busy to allocate enough time to educational affairs	HR13		New tools & technologies are not used in patient education	E13
	The nurse emphasizes clinical practices more than education	HR14	Challenges related to management and system (M)	Lack of effective interaction during education and lack of patient involvement	M1
	In face-to-face education, it is not possible to supervise the education process by a qualified person. The confirmation form of education is completed by the nurse; therefore, it is impossible to make sure of transmitting all the necessary information	HR15		Incomprehensive and incomplete educational protocols (the nurse authority is restricted to transmitting clinical information by protocols and rules)	M2
	The nurse authority is limited in transmitting some educational information	HR16		The patient's mental status is not considered during the education	M3
	Some nurses have low communicational skills	HR17		Patients are not grouped based on their physical weakness and disability	M4
	Different types of treatment and the possible complication are not explained by the medical team to the patient	HR18		Due to a lack of pre-awareness in the outpatient treatment and hospitalization stage, the patient does not have enough physical & mental readiness	M5
	The medical team does not provide an explain while prescribing aggressive procedures	HR19		The patients who do not attend the educational classes due to their specific conditions or period of the treatment will lose group education	M6
	Oral education is not presented by the medical team during the discharge	HR20		There is no rule for disambiguation and pre-procedure education for non-invasive procedures	M7
	In the surgery department, there is less educational communication between the surgeon or her/his assistants and patient	HR21		The focus of the evaluation system is more on writing reports than on clinical practices and communication between nurses and patients	M8
	The experience & educational skills of doctors are different and, in some cases, inadequate	HR22		There are no good plans for the patients’ other diseases	M9
	Being too busy, the doctor cannot allocate enough time to talk with the patient	HR23		The education of preventing frequent disorders in ICU is not sufficiently considered	M10

**Fig. 3:**
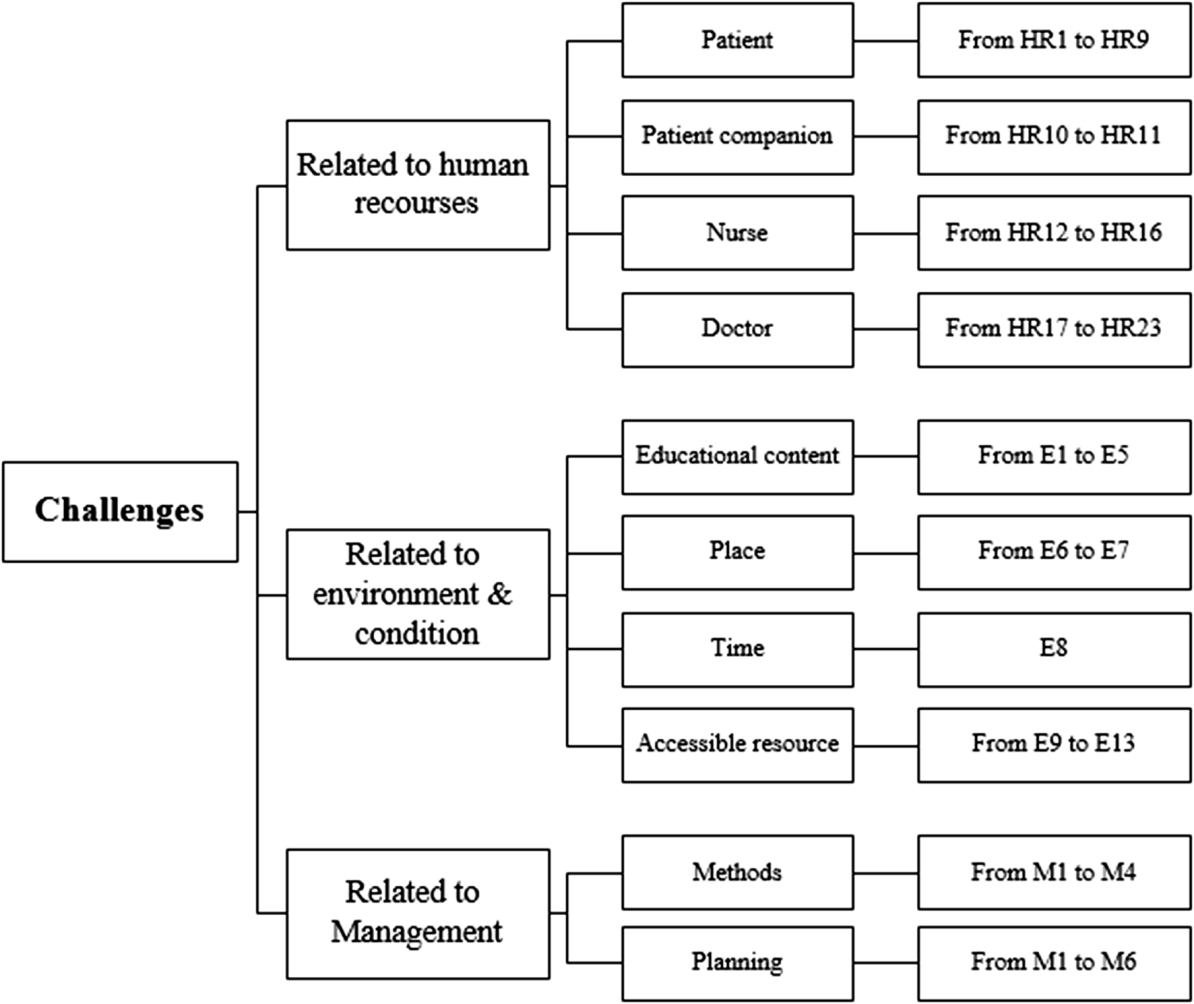
Classification of the challenges of patient education

Figure 3 shows the challenges identified in the hospital under study for cardiovascular patients. As indicated, they have been grouped into three main categories and several sub-categories after interviewing the hospital experts. The categorization given in Fig. 3 reveals which of the challenges extracted from interviewing the experts falls into which of the categories in Table 1.

After identifying the challenges, their prioritization is vital because there are too many challenges with varying degrees of importance, so considering them as equal is irrational. As a result, in the next step, prioritizing the challenges of the patient education process is done.

### Prioritizing the challenges of the patient education process by PROMETHEE II method

In this stage, the challenges of the patient education system have been prioritized using the PROMETHEE method. Using this method, several factors involved in the education process were simultaneously considered, and making use of different mathematical relationships; their total impact on the options was determined. Thus, a collection of criteria involved in the education process were extracted from the previous articles. Next, asking the hospital experts, some extracted criteria were removed, and some were modified and adjusted to be tangible to the hospital scores.

For instance, in Table 3, the first criterion (patient knowledge) and the second criterion (patient's attitude and belief) were derived from Boyde et al. [6]. The third, fourth, and fifth criteria were determined based on the hospital experts' opinions. Eventually, the experts' points of view on these five criteria were aggregated (presented in Table 3). Then using the five final criteria, the challenges were prioritized with the help of the PROMETHEE II method.

**Table 3:** Selected criteria from the research background to prioritize the educational challenges

Criterion	The meaning of the criterion in the health context	Description
1. Knowledge	Effective on understanding & learning the content	This criterion indicates how much the challenge or the factor will affect the patients' learning process. Five-point Likert scale (1 for completely agree and 5 for completely disagree) is used
2. Attitude & belief	Effective on observing the principles of self-caring and motivation	This criterion shows how much the factor will affect motivating the patients and persuading them to cooperate in education issues. A Five-point Likert scale (1 for agree completely and 5 for disagree completely) is used
3. Wastage	Waste of personnel’s time & energy	This criterion shows how much this factor will affect the personnel’s time and energy wasting. A Five-point Likert scale (1 for agree completely and 5 for disagree completely) is used
4. Probability of occurrence	Probability of occurrence	This criterion explains the probability of the disorder occurrence. Five-point Likert scale (1 for occurrence and 5 for less occurrence) is used
5. Overall effect	Effect on the patient's recovery and treatment	This criterion indicates how much the effective factor will affect the patient's treatment process. A Five-point Likert scale (1 for agree completely and 5 for disagree completely) is used

In this method, to obtain the decision matrix points, scoring forms were filled-out with the aid of 17 hospital experts and 15 patients from the same hospital. The experts included nurses and doctors working in different internal and surgical departments with at least six months of experience in that ward interested in participating in this study. They included three general practitioners, one quality manager, seven head nurses, and six nurses.

After extracting information from the collected forms, the decision matrix was created, and for simplicity, the weight of all criteria was assumed to be the same (w = 0.2). Due to the large content of the decision-making matrix and computational complexity, they were discarded. Then, using the decision-making matrix and the weight of criteria, the PROMETHEE II method was applied according to the V-shape function [23] with indifference area. It is to be noted that MATLAB software was used to prioritize the challenges. The values of Φ and the ranking of the challenges are presented in Table 4.

**Table 4:** Prioritizing the challenges of the education process through the PROMETHEE II method

Challenge code	Total Φ	Challenge rank	Challenge code	Total Φ	Challenge rank
HR13	0.0378	1	E3	0	24
HR2	0.0199	2	M2	0	25
HR1	0.0192	3	HR10	0	26
HR11	0.0191	4	M10	0	27
HR5	0.0085	5	HR4	0	28
HR3	0.0081	6	E10	0	29
HR8	0.0043	7	M7	0	30
E6	0.0041	8	E9	0	31
E13	0.0036	9	M1	0	32
HR17	0.0026	10	HR23	0	33
HR21	0.002	11	E3	0	34
HR9	0.002	12	HR19	0	35
HR6	0.0016	13	E4	0	36
E7	0.0016	14	HR18	0	37
E9	0.0015	15	HR20	0	38
M8	0.0015	16	HR14	− 0.0007	39
E12	0.0011	17	HR16	− 0.0016	40
M6	0.001	18	HR12	− 0.0018	41
M9	0.0005	19	HR22	− 0.0025	42
E5	0.0005	20	HR15	− 0.0028	43
M3	0	21	E1	− 0.0055	44
M5	0	22	E11	− 0.0131	45
M4	0	23	E8	− 0.0179	46

According to Table 4, The first five items of prioritizing the challenges were as follows:

1. The nurse is too busy to allocate enough time to educational affairs.
2. The patient does not understand the meaning of education content.
3. The patient does not listen carefully (The patient does not follow the educators & does not pay due attention to the educations).
4. Some of the patient's caregivers request for the same education that has already been given to the patient.
5. The patient does not understand the standard language.

As shown in Table 4, among the top 10 challenges, most challenges are related to human recourses (80%), and in this group, the biggest proportion goes to patients (50% of the whole challenges). After that, nurse-related challenges are the second human factor with a share of 20%. The environment and condition group, having two options in the 8th and 9th ranks, is the second group among the three main groups (human resources, environment & condition, and management).

### Identifying the root causes of the challenges of the patient education process

After clarifying the priority of the challenges and selecting the top ten challenges, it is necessary to detect their root causes by hospital experts to be able to suggest improvement scenarios for the education system.

To implement this stage, after selecting ten risky challenges, again with the help of hospital experts who participated in the challenges identification phase, in-depth semi-structured interview sessions were separately held in the hospital’s meeting room to discuss and identify the root causes of these challenges. The experts participating in this phase included eight nurses, three physicians, and one hospital manager. In the interviews, each interviewee was asked two basic questions about each challenge:

- What are the reasons for these challenges (depending on their area of expertise)?
- Which actor in the education system is the main cause of each challenge?

The interviews were conducted separately in the meeting. Then all answers to the above questions were collected and then coded. Similar responses were assigned the same codes. Then, with the help of content analysis, cause-and-effect diagrams (Ishikawa diagram) were drawn for each challenge. Finally, these diagrams were verified by all the interviewees, and corrective action was taken, if necessary. Table 5 shows the root causes of the challenge of educating patients.

**Table 5:** Root causes of the challenges of educating cardiovascular patients

The challenges	Root causes of the challenges	
**HR13:** The nurse is too busy to allocate enough time to educational affairs	Personnel	Lack of time management Incorrect perception of education Lack of motivation No extra financial bonus for holding educational classes
	Lack of human resource	Lack of allocated budget Inefficient management decisions
	Ineffective methods	No group education classes by the nurses No repetition of training during presenting clinical services
**HR2 & HR5:** The patient does not understand the meaning of education content, and the patient does not understand the standard language	Learner	Low literacy Old age Linguistic and cultural differences
	Educator	Poor expression skills Lack of work interest Poor teaching skills
	Educational tools	Difficult language of written educational tools Lack of diversity and attractiveness
**HR1:** The patient does not listen carefully and does not accompany the educator	Learner	Not being interested in learning Unfavorable physical and mental status Not knowing the importance of education
	Educator	Not paying due attention to the patient Little experience Poor expression skills Weak personal characteristics No extra financial bonus for holding educational classes Lack of motivation
	Educational tools	Lack of diversity and attractiveness Inaccessible during the classes Lack of interactive multimedia tools
**HR11:** Some of the patient's caregivers request for the same education that has already been given to the patient	caregivers' education	caregivers' education is not considered important according to lack of a clear plan for involving them in the education
	Educational tools and caregivers	Lack of educational tools Not distributing the available sources among all caregivers present in the wards
**HR3:** The patient forgets	Education	Not repeating and reviewing for the patient Not evaluating the patient Difficulty in memorizing the education content
	Educational tools	Lack of diversity and attractiveness Inaccessible during the classes Lack of interactive multimedia tools
	Learner	Age Mental status Physical status
**HR8:** The patient does not accept the illness, or the mental problems resulting from the illness decrease the education effectiveness	The patient's mental condition is not considered	
	There is no mechanism for the initial evaluation of all patients' mental condition	
	New tools and technologies are not practically used in patient education	
**E6:** The education room or the inpatient ward room is small, and there are not adequate group classrooms	Patient status	Impossibility of moving the patient to another room
	Available space	Lack of suitable space
**E13:** New tools & technologies are not used in patient education	The current system (Internet Radio)	Lack of a suitable notification system for patients Not being attractive and dynamic
	Management	Lack of infrastructure Insufficient financing Management not being familiar with new educational tools
**HR17:** Some nurses have low communicational skills	Educator	Little experience Weak personal characteristics Poor expression skills
	Management	Insufficient in-service training Lack of organizational commitment due to frequent relocation of personnel

The top 10 challenges are written in the main section of Table 5 (first column). The related causes are mentioned in the second column, and each subject's characteristics are stated in the third column. While studying the root causes of the challenges, it was found that there were commonalities in the causes of the occurrence of the second to ninth challenges, mainly in the patient group. These commonalities are:

- Root causes related to the learners, including low literacy, old age, linguistic and cultural differences, unfavorable physical and mental status, not being interested in learning, and not being aware of the importance of education in the treatment process.
- Root causes related to the educators, including poor expression skills, personal characteristics, incorrect perception of education, lack of work motivation and experience, and improper time management.
- And root causes related to educational tools, including lack of simplicity, diversity and attractiveness, inaccessibility, and lack of interactive multimedia tools.

**Fig. 4:**
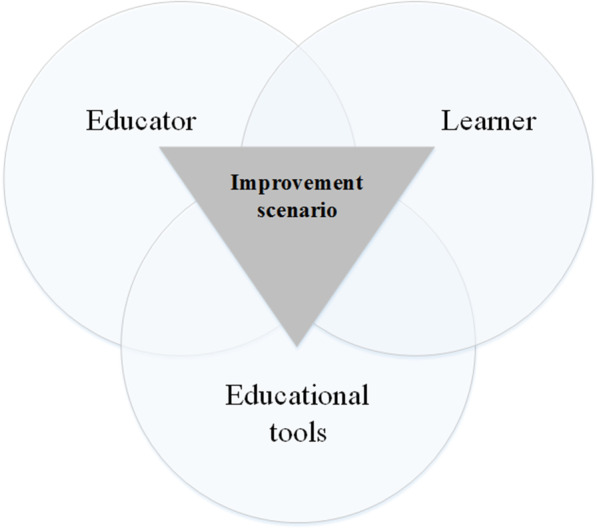
Combination of the three repeated root causes of challenges

Examining the challenges and their root causes shows that in the challenges that the patient is the main factor, other factors are also effective. Many patients refuse education, but it is clear that the causes of disorders related to patients are mainly out of their control. Accordingly, solving any of these challenges alone would not be sufficient and effective to improve the whole system. On the other hand, the implementation of some improvement suggestions may affect other conditions due to the complicated causal relationship, which makes it necessary to develop a more general framework. As shown in Fig. 4, while providing improvement solutions, these factors (educator, learner, and educational tools) need to be taken into account interactively. Consequently, it has been tried to provide a suitable and practical framework by combining different ideas by considering the condition of the educational system and the existing facts.

Accordingly, and based on the top five challenges analyses (root causes), a set of improvement suggestions for these challenges were presented. For the first-ranked challenge (HR13), in which "heavy nursing workload" prevents allocating enough time to patient education, employing new experts and experienced personnel called "tutors" was suggested; they can follow and evaluate both learners and educators. In this way, the nursing workload will be reduced. Along with the routine and implicit training through patient-nurse conversations, the education classes will be managed by the tutor in an appropriate manner and on time. In addition, according to the root causes of the second-ranked and third-ranked challenges (HR2 & HR1), which refer to their inability in understanding the education content, a few shared solutions were presented as follows:

- Designing and implementing in-service training to promote organizational culture, expression skills, and effective communication,
- Patient mental screening at the time of entry to the hospital, and recording it for effective use of psychiatric services,
- Emphasis on the companionship in the case of unfavorable mental status,
- Providing optometry and audiology services for patients, especially the elderly, and then archiving their documents.

For the fourth-ranked challenge (HR11), which implies a deficit caused by the patients' caregivers, some suggestions were made as follows:

- Determining a mechanism for educating patient's caregivers
- Providing an educational poster with common questions and their answers in two copies (one for caregivers and the other for pasting in the hospital room)
- Preparing a poster containing common questions of CVDs with answers to the patients.

Finally, for the fifth-ranked challenge (HR5) that again refers to the patient's weakness in understanding the standard language, the suggestion is that the presence of a caregiver should be mandatory for these patients, or a person who understands the patient's language completely (translator) should be quickly called. Nurses should also be advised during the in-service training courses to speak slowly and fluently with the patients.

### Designing a framework for essential challenges in the training processes using the proposed scenarios

**Fig. 5:**
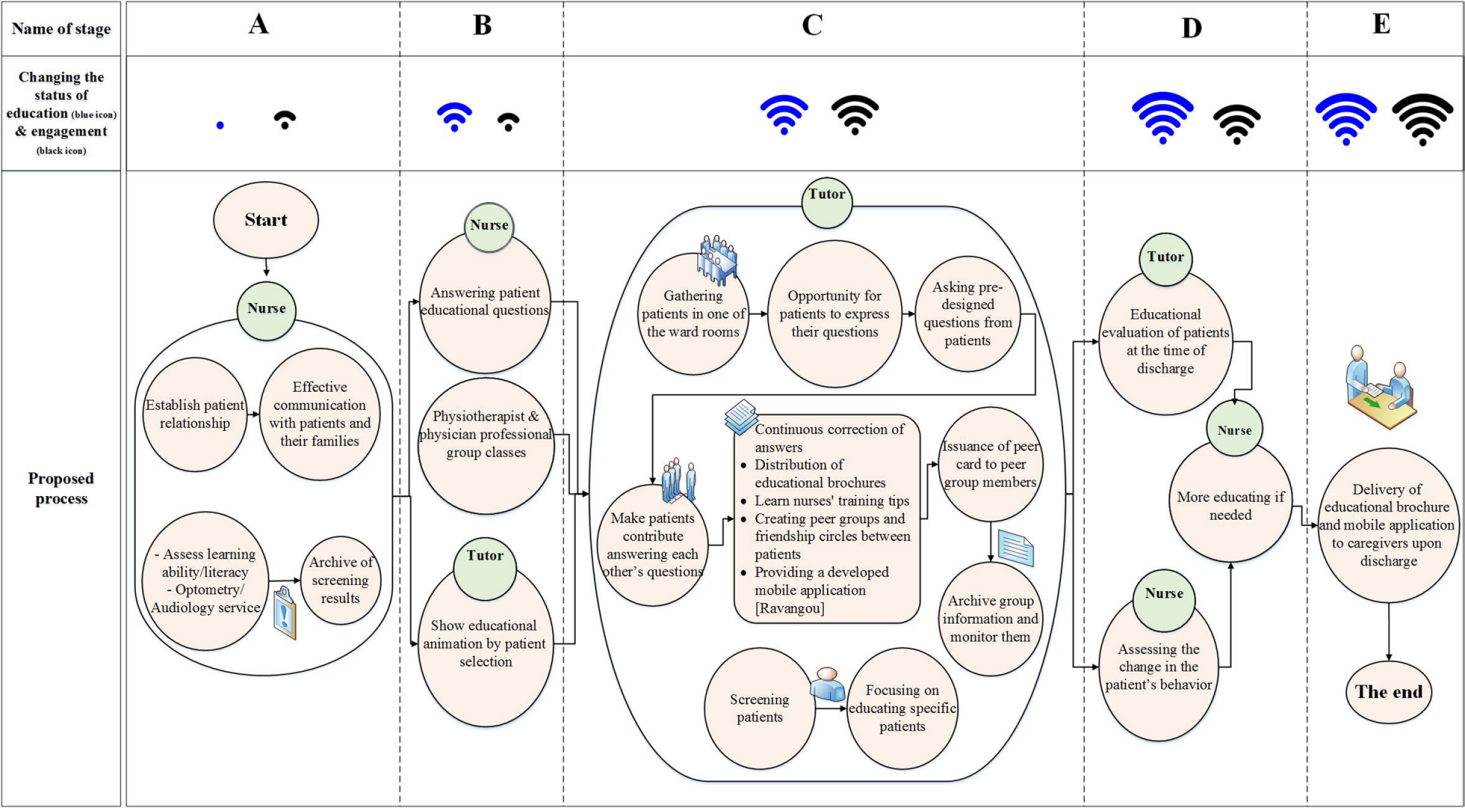
Proposed framework for the process of education and engagement of cardiovascular patients

In the final step, using the findings of the present research and focusing on the challenges affecting the education system, a framework improving the education processes was designed with several hospital experts, including THC educational supervisor and two doctors. The final developed patient education framework is illustrated in Fig. 5. Before implementing it, a series of prerequisite measurements should take as follows:

- All nurses participate in in-service training about organizational culture, effective communication, and expression skills.
- An experienced specialist person called "tutor" is employed to follow and evaluate the learners and educators.
- Some brochures and software are designed and introduced, which are useful tools from this plan's point of view. The software includes a training program in Persian with a straightforward user interface, consisting of videos and animations displaying the mechanism of cardiovascular system function, frequently asked questions and answers about CVDs, and some tips for each group of patients. This software can be run on Windows and Android OS, smartphones, and smart TV.
- During the education, if the patient cannot participate in the educational activities, his/her caregiver can participate instead or together with the patient.
- Peer education means pairing and grouping patients so that they can follow each other's learning. These groups can help to motivate patients and increase their self-esteem by creating friendship rings.
- The friendship groups can also connect rehab groups or even work separately. For example, people with similar diseases use social networks or chat rooms to become aware of each other's healing process and other therapeutic activities. These groups can also be run under the supervision of the tutor.

## Discussion

In the present study, a framework for cardiovascular patients' education (shown in Fig. 5) has been designed and presented based on analysis of the results of ranking the challenges (Table 4) and their root causes (Table 5). The framework consists of five components, which are shown by A-E Latin letters in Fig. 5. In these components, the effort has been made to completely cover the process of receiving educational services by cardiovascular patients. In the framework, solutions are provided for the overwhelming challenges recognized as important in the prioritization operation with the PROMETHEE II method. The following five components are described:

- **Component A:** The framework is started with the patients' entrance to the department, and they experience the first encounter with the nurses. In component A, some solutions are provided based on initial nurse-patient interaction and communication. As mentioned, nurses' behavior, conversations, and communications in human resources-related challenges have a significant impact on educating patients (10th rank of the challenges is related to how nurses communicate with patients (HR17)). Besides, in the second-ranked challenge (HR2: The patient does not understand the meaning of education content), third-ranked challenge (HR1: The patient does not listen carefully), and fifth-ranked challenge (HR5: The patient does not understand the standard language), it is necessary to identify the patients’ weaknesses, and then perform the screening of factors affecting the education, like the patients’ hearing, vision, literacy, and language status. The proposed framework provides solutions in the field of educational tools and educators to the mentioned challenges. Simplification of written instruments and the use of visual and attractive tools ("Ravangou" animations) are among these solutions. In addition, peer education and creating a friendly question–answer environment by the tutor allow for more education for patients who do not understand the concepts or do not follow the education.
- **Component B:** A new educational assistant named “tutor” enters into the process and provides stabilizer tutorials using new educational tools according to the patient choices alongside with the physicians’ and physiotherapists’ professional group educations that are already in progress. These actions are in line with the third-ranked, sixth-ranked, and ninth-ranked challenges (HR13, HR1, HR3, and E13). In this component, the challenges of the high workload of nurses, the failure to accompany the patient during education, forgetting the education content, and not using the modern educational tools have been addressed, respectively. It is worth noting that HR13 is the first-ranked challenge refers to the nurses' lack of time, which the lack of enough personnel and inefficient methods are its root causes. In this regard, two solutions are presented: (1) grouping education and dividing the various education points that should be educated by the nurses between different intervals and (2) introducing a tutor who has enough expertise and experience in education. The tutor will eliminate the cost of hiring a new nurse.
- **Component C:** The tutor plays a critical role and takes many responsibilities alone, such as organizing groups to clarify the ambiguities and concerns that might come to the patient's mind during or after education but have not been answered for various reasons. In these group meetings, a mobile app called "Ravangou" is added to the previous educational tools in response to the 9th challenge (E13: New tools & technologies are not used in patient education). The tutor can form friendship circles and peer teams to engage the patients in each other's education and motivate them by donating a peer card. This solution is suggested to improve seventh-ranked challenge so that the patients can overcome their mental problems by being in friendship circles. Meanwhile, the tutor the supervisor focuses on educating patients with specific conditions based on the nurses' primary screening list.
- **Component D & E:** In component D, patients and their caregivers are evaluated for the training's effectiveness. Finally, in component E, the FAQ brochure and mobile application are also delivered to the patient's caregiver. This solution is included in the proposed framework to overcome the fourth-ranked challenge (HR11: Some of the patient's caregivers request for the same education that has already been given to the patient). In addition, the caregiver’s presence and providing the educational content for them, are considered as a solution to the fifth challenge (HR5: The patient does not understand the standard language). As a result, the proposed solution is to accompany patient caregivers in education.

In the present study, we have provided a novel framework to describe the processes and the interactions between the different actors (patients, nurses, and tutors). The solutions presented in this framework are a combination of industrial engineering-based solutions (e.g., adding a new specialist workforce called tutor), IT-based solutions (e.g., "Ravangou" Mobile App), and learning-based solutions (e.g., improving communication, health literacy, and so on). Each of these solutions is based on a root cause analysis of the challenges in the system.

This study is prerequisite research for proposing improvement tools. In some studies of this area, due to the lack of such preliminary studies and the lack of proper understanding of system challenges, adherence to the tools suggested may decrease over time. Using this method, hospitals can continuously be informed of the bottlenecks of their education system and also significantly be raised their awareness about the as-is processes to support the educational system improvement.

It is worth mentioning that the proposed framework has been delivered to the experts (including THC educational supervisor and two doctors) in the hospital under study, and their correcting ideas have been applied to the framework and eventually confirmed by them. In fact, to evaluate the conceptual framework, we have used the member checking technique [24, 25] in which the participants themselves have examined the researchers' proposed model and then validated it.

Previous research in the field of health education systems analysis have often used purely qualitative methods in their studies [7, 9, 10, 12–14, 17, 26]. In contrast, we have used a combination of qualitative and quantitative methods that can improve the results by guaranteeing that the limitations and weaknesses of one type of approach are balanced by the strengths of another. Indeed, ‘mixed-methods’ represents nowadays a rapidly developing field of health science methodology. In practice, most researchers agree that “mixed-methods” produces a richer and more comprehensive understanding of a research area. Therefore, the approach used in this research should be considered as a new step in the innovative combination of qualitative and quantitative methods to the health education area. The achievement of this study is valuable for various reasons, including the difficulty of conducting quantitative research in the field of health education, and can be the starting point for using this research method in future studies.

Another feature that distinguishes this study from similar researches is its comprehensive view of educational processes and their related challenges. This point has been confirmed several times by the hospital experts. In addition, using multi-criteria decision-making methods like “PROMETHEE” is new in prioritizing the challenges of patient education and engagement. Besides ranking, its steps are simple and understandable for all stakeholders involved. With the above explanations, the strengths of this study can be summarized as follows:

- Providing a framework with applying different quantitative and qualitative methods (mixed-method approach), including BPM, semi-structured in-depth interviews, multi-criteria decision-making, and root causes analysis to improve processes in patient education.
- Presenting a novel framework to characterize the process and the interactions between the different actors (patient, nurses, and tutors)
- Highlighting the crucial role of educators in the education processes as they can reinforce the patient’s ability to engage in the education processes. This study also indicates the importance of patients’ health literacy in educational systems.
- A better understanding of the roles of educators, learners, and educational tools in managing cardiovascular patients may be valuable for translating this study’s implications to other chronic diseases.

However, there were some limitations in conducting this study. The research was conducted in only one hospital, although the research method used is applicable in other hospitals and chronic diseases with minor modifications. In order for the proposed framework to be generalizable, we need to implement it in the education system of some other hospitals and compare the results. As a result, the results are not generalizable without additional research. Some of the solutions suggested in this framework might seem impossible to be fulfilled in other hospitals, but these can be customized to a specific hospital's atmosphere.

## Conclusions

In this study, a framework for improving the education processes of cardiovascular patients was presented. We concluded that the workload of nurses and their lack of time is the most important challenge in the patients' education processes. This conclusion is consistent with the results obtained in the research background. A literature review shows that the challenge of "personnel's time shortage and time-consuming patient education" has been one of the most important challenges identified in previous studies.

In future research, the scenario of adding new workforces (both nursing or non-nursing staff) can be simulated, and the results can be evaluated. It is also possible to apply a process-mining approach to improve process efficiency and understanding of processes and obtain the possible causes of the challenges by focusing on more detailed education process components. On the other hand, as the processes have been drawn up using the BPMN standard, they are easily understood by process designers and developers; hence they can be the basis of the development of IT tools in health-related areas.

From another perspective, the challenges can be reviewed using a fuzzy cognitive mapping to provide the possibility of identifying the factors affecting the educational process. This method enables the decision-maker to have a good understanding of the causal relations between the factors and the relative direction and strength of the relations between them.

## Supplementary Information

**Additional file 1: Title of data: A.1.** Description of data: The main processes of education in the inpatient ward.

**Additional file 2: Title of data: A.2.** Description of data: The main processes of education in the surgical inpatient ward.

**Additional file3: Title of data: A.3.** Description of data: The main processes of education in the angiography department.

## Data Availability

The datasets analyzed during the current study will not be publicly available because the present article is the result of a master's thesis and its graduation process has not been completed yet. According to the regulations of *Tarbiat Modares University*, authors are not permitted to share data until graduation processes are completed. The data of the present study will be available by the corresponding author on reasonable request after the completion of graduation processes.

## Abbreviations

CVDs: Cardiovascular diseases
MCDM: Multiple criteria decision-making
WHO: World Health Organization
CH: Challenge
THC: Tehran Heart Center
BPM: Business Process Management
BPMN: Business Process Model and Notation
PROMETHEE: Preference ranking organization method for enrichment evaluation
