# Supplementary figures and images for: A novel approach to educate hospitalized cardiovascular disease patients about lifestyle and behavior modifications
Source: BMC Med Inform Decis Mak. 2021 Nov 20;21:321. doi: 10.1186/s12911-021-01680-x (PMC8606092; doi:10.1186/s12911-021-01680-x)

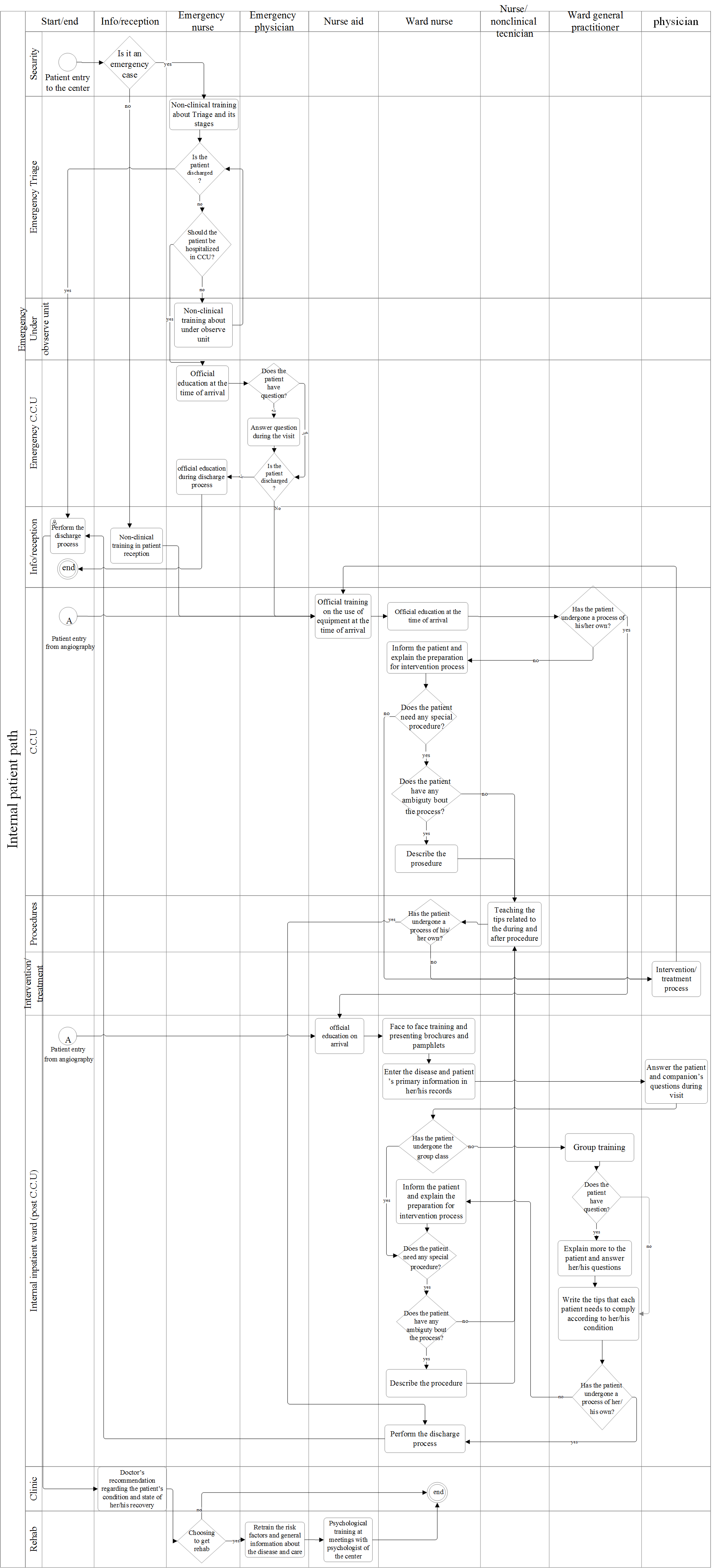

Supplement: Supplementary file 1 — Additional file 1: Title of data: A.1. Description of data: The main processes of education in the inpatient ward. [file 12911_2021_1680_MOESM1_ESM.tif]

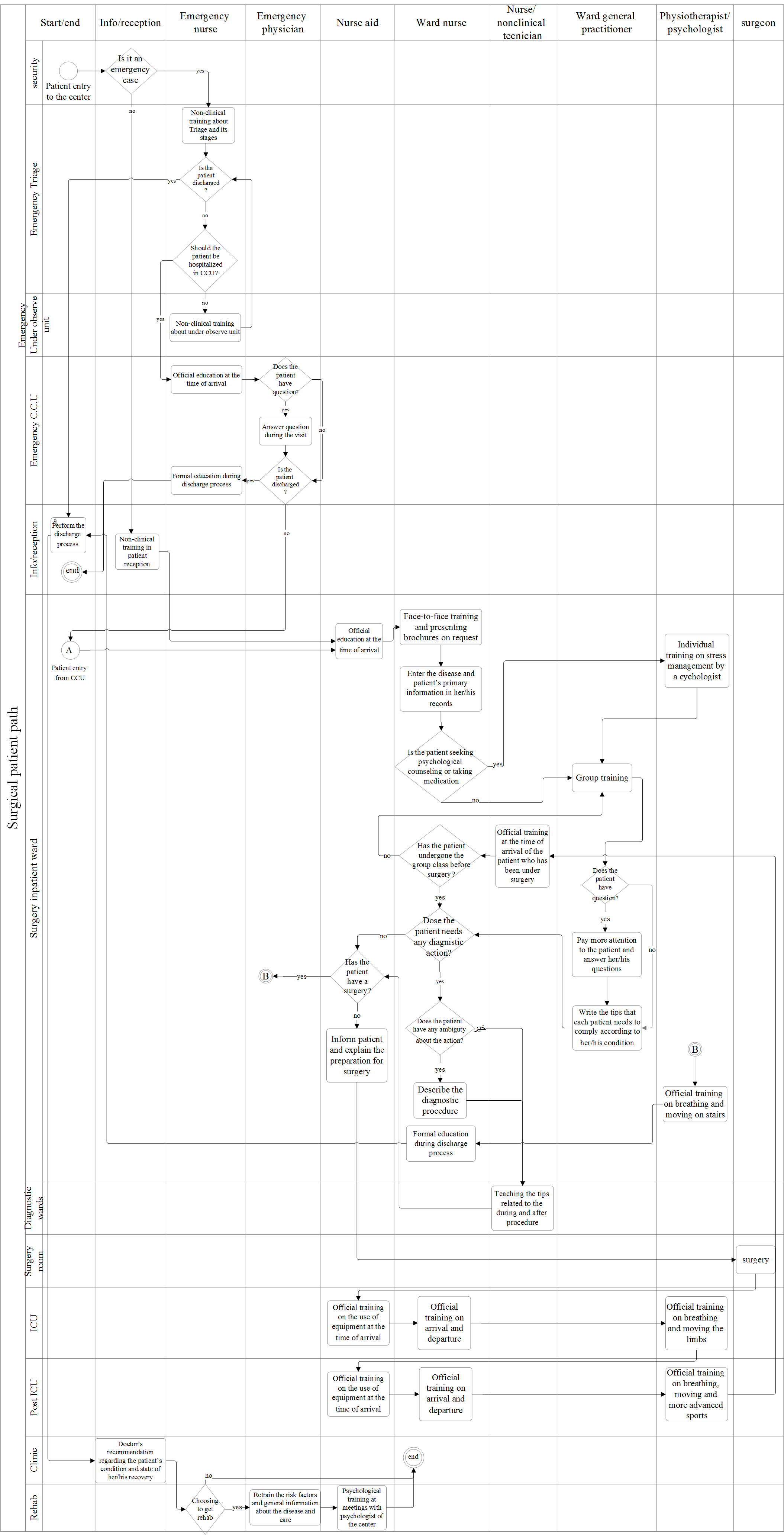

Supplement: Supplementary file 2 — Additional file 2: Title of data: A.2. Description of data: The main processes of education in the surgical inpatient ward. [file 12911_2021_1680_MOESM2_ESM.tif]

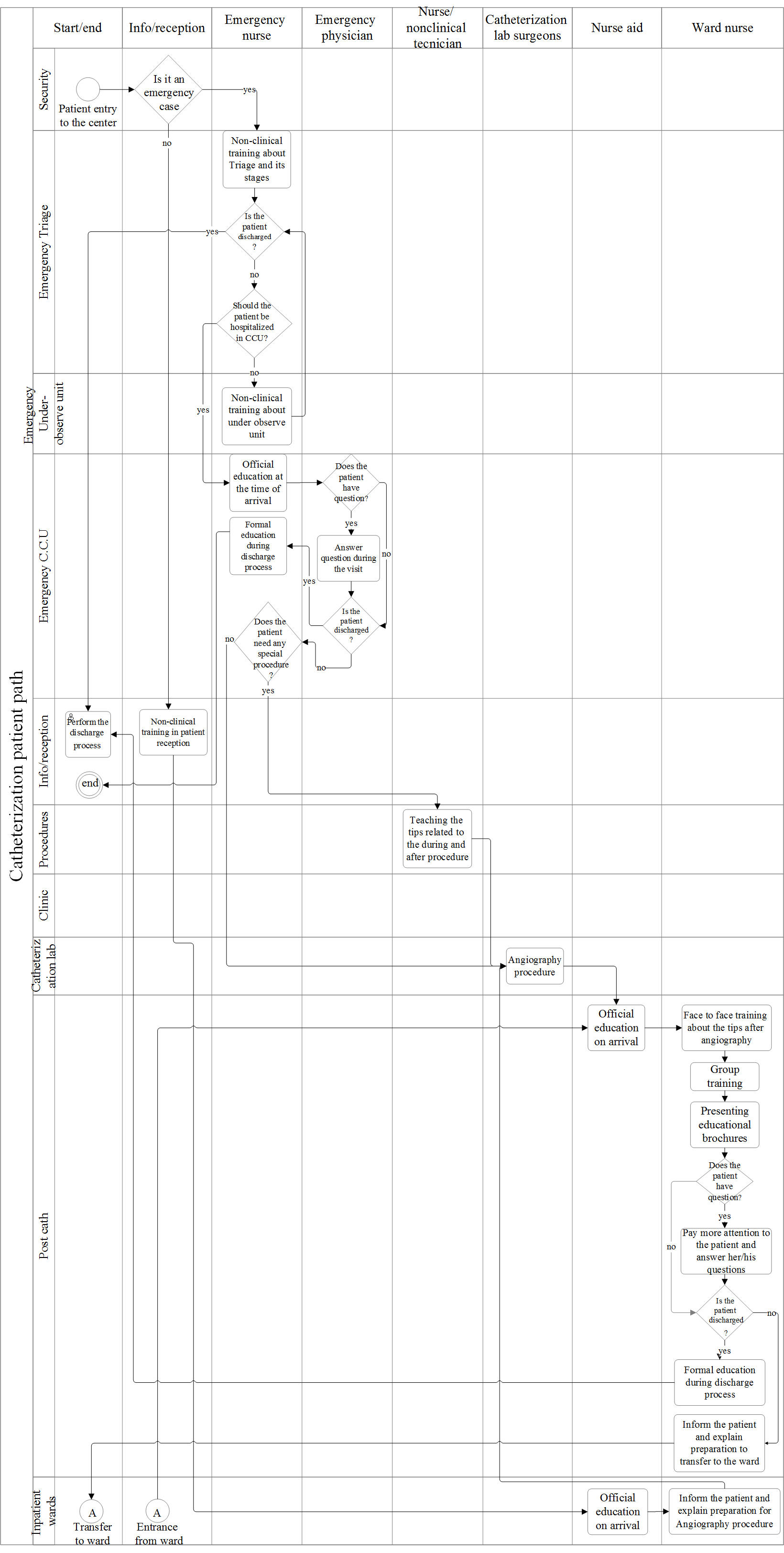

Supplement: Supplementary file 3 — Additional file3: Title of data: A.3. Description of data: The main processes of education in the angiography department. [file 12911_2021_1680_MOESM3_ESM.tif]
